# Production of *icaADBC-*encoded polysaccharide intercellular adhesin and therapeutic failure in pediatric patients with staphylococcal device-related infections

**DOI:** 10.1186/1471-2334-10-68

**Published:** 2010-03-15

**Authors:** Bernardo Diemond-Hernández, Fortino Solórzano-Santos, Blanca Leaños-Miranda, Leoncio Peregrino-Bejarano, Guadalupe Miranda-Novales

**Affiliations:** 1 Departamento de Pediatría, Hospital de Infectología, Centro Médico la Raza, Mexico City, Mexico; 2 Departamento de Infectología, Hospital de Pediatría, Centro Médico Nacional Siglo XXI, Instituto Mexicano del Seguro Social, Mexico City, Mexico; 3 Unidad de Investigación en Epidemiología Hospitalaria, Coordinación de Investigación en Salud, Instituto Mexicano del Seguro Social, Mexico City, Mexico

## Abstract

**Background:**

Biofilm production has been established as a virulence factor which allows *Staphylococcus *to adhere and persist in medical devices. The objective was to determine whether therapeutic failure in patients infected with *Staphylococcus *spp. is linked to biofilm production, the presence of the *ica *operon, and the bacterial insertion sequence element *IS*256.

**Methods:**

*Staphylococcus *spp. isolates from patients with device-related infections were collected. Therapeutic failure with proper antimicrobial treatment was registered. Biofilm phenotype was determined by Congo red test agar and Christensen assay. Presence of the *ica *operon genes *A-D *and *IS*256 was detected by PCR. Differences were compared through *x*^2^.

**Results:**

100 isolates from staphylococcal infections episodes were included: 40 sepsis/bacteremia, 32 ependymitis, and 28 peritonitis. 73.77% of CoNS and 79.5% of *S. aureus *isolates harbored the *icaD *gene, 29% of all isolates *IS*256-A+ *IS*256-D genes, *icaA *and *icaB *genes were only found in CoNS (27.8% and 21.3% respectively). Therapeutic failure occurred in 95.4.% of patients with a positive *IS*256-A+ *IS*256-D *S. epidermidis *isolate, RR 5.49 (CI 95% 2.24-13.44 p ≤ 0.0001), and 85.76% in CoNS isolates, RR 2.57 (CI 95% 0.97-6.80, p = 0.05). Although none *S. aureus *was positive for *IS*256-A + *IS*256-D, therapeutic failure was observed in 35.8%.

**Conclusions:**

The presence of *icaA/D *genes along with the sequence element *IS*256 was associated with therapeutic failure in most CoNS infections, even though its absence in *S. aureus *isolates does not ensure therapeutic success.

## Background

Staphylococci inhabit the skin and mucous membranes of animals and humans. Due to the ability to produce biofilm, they are also a common cause of device-associated infections [1-4]. Biofilm formation capacity is associated with antimicrobial resistance, and considered widely as a virulence factor. Invasive isolates are more prone to produce biofilm than carriage isolates of healthy individuals [5,6]. *Staphylococcus epidermidis *was the first species to be described as a biofilm producer; however, the same ability is encountered in *S. aureus *and other coagulase-negative *Staphylococcus *species [7,8].

The principal component of biofilm is a polysaccharide intercellular adhesin {PIA} [9,10]. PIA is composed of a beta-1,6-N-acetylglucosamine polymer synthesized by an enzyme codified by the *ica *operon found on the bacterial chromosome, that includes a regulating element of four genes (*A, B, C*, and *D*), and a transposable element, *IS*256 [11]. It is known that the *A *gene codifies the N-acetylglucosamyl transferase enzyme responsible for synthesizing PIA. This enzyme is not very active *in vitro*, but co-expression of the *D *gene increases the activity. *IcaB *is the deacetylase responsible for the de-acetylation of mature PIA and the transmembrane protein *IcaC *seems to be involved in externalization and elongation of the growing polysaccharide [9,12].

The expression of the *icaADBC *genes is controlled by a complex variety of conditions and factors; one of them is the excision or insertion of the bacterial sequence element IS*256 *at various locations on the operon. The molecular basis for the regulation process is still unclear; however, the insertion element is definitely responsible for up to 33% of the activated portion of the operon that allows its expression [1,10,13,14]. Other regulator genes (*Rsb*U, σ^B^, *Tca*-R, *agr, sarA*) for PIA production have also been reported [15].

Biofilm production usually occurs in two steps, 1) the initial step of adhesion to the surface, facilitated by adhesive polysaccharides and/or a polysaccharide with multiple proteins (including autolysins), and 2) the accumulation of cells due to PIA production, synthesized after activation of the *ica *operon [9]. Initial findings assumed that the presence of ica*ADBC *genes will contribute to the persistence of infection and therapeutic failure in presence of a medical device. Some authors have tried to establish the presence of the *AD *genes as a prognosis biomarker in device associated infections [8,16]. Many other environmental conditions and the phenotype of the *Staphylococcus *isolate also participate in the regulation of biofilm production [17]. By understanding the different mechanisms of biofilm production it will be possible to support the development of therapeutic strategies [5].

The objective of this study was to describe the association between biofilm production, the presence of *icaADBC *genes, the bacterial insertion sequence IS*256 *and therapeutic failure, in isolates from patients with medical device-related infections.

## Methods

Clinical staphylococci isolates were collected prospectively from hospitalized patients with a staphylococcal medical device-related infection from September 2002 to July 2003. Isolates were obtained from blood, cerebrospinal fluid and peritoneal fluid. The protocol was approved by the Institutional Review Board. Patient's data were obtained from clinical records. Infection was defined in accordance with internationally proposed criteria [18].

Empirical antimicrobial treatment was prescribed for all patients, and was modified according to susceptibility patterns.

Therapeutic failure was considered:

1) For central venous catheter (CVC) related bacteremia/sepsis, persistence of positive blood cultures collected through the CVC after 72 hours of administering proper antimicrobial treatment.

2) For peritonitis, persistence of positive peritoneal fluid culture collected through peritoneal dialysis catheter or a cellularity ≥ 100 cells per mm^3 ^after 72 hours of administering proper antimicrobial treatment.

3) For ependymitis, after removal of the infected shunt, persistence of positive cerebrospinal fluid (CSF) culture collected through external ventriculostomy, after 72 hours of systemic and/or intraventricular antimicrobial treatment.

### Microbiology

*Staphylococcus *species were identified by API-Staph system (bioMérieux). Antimicrobial susceptibility was determined in accordance with CLSI recommendations, oxacillin resistant isolates were tested for *mec*A gene by PCR [19,20].

The isolates were characterized phenotipically by culture on Congo red agar plates (CRA) as described by Freeman et al. [21], with modifications as described by Arciola et al [22]: agar plates were prepared with 0.8 g of CRA (Sigma) and 36 g of saccharose (Sigma) to 1 liter of brain heart infusion agar (Dibico, Mexico) and incubated 24 h at 37°C and subsequently overnight at room temperature. For *S. aureus *CRA were kept up to 72 h.

The quantitative assay by Christensen was used to test the ability to produce biofilm [23]. Briefly, 1:100 dilutions of overnight cultures in trypticase soy broth were used to inoculate wells in a microtiter polystyrene plate (Falcon, Becton Dickinson, Labware, NJ, USA). After incubation for 24 h at 37°C, the plates were gently washed two times with phosphate-buffered saline (PBS, 10 mM potassium phosphate, 0.15 M NaCl pH 7.0), and stained with 1% (w/v) crystal violet solution; the excess stain was washed off with demineralised water. The adherent cells were resuspended in acid-isopropanol (5% v/v 1 M HCl in isopropanol), and the absorbance (A) was measured at 492 nm in a microplate reader. A biofilm producer strain was defined as an optical density at 492 nm of ≥ 0.17. Clonal relatedness of strains was excluded using Pulsed Field Gel Electrophoresis (PFGE) [24].

The presence of the *A, B, C*, and *D *genes of the *ica *operon and the position of the bacterial sequence element IS*256 *were detected by PCR according to the protocol described by de Silva G. [6] with some modifications: DNA extraction was performed with the Promega^® ^Wizard Genomic Kit (Madison, WI, USA). Primer sequences were taken from the GenBank sequence database of the National Center for Biotechnology Information [GenBank accession number U43366 for *S. epidermidis*, EF546621 for *S. lugdunensis*]. The sequence for the bacterial insertion element was taken from the publication by Ziebuhr [25].

The PCR cycling conditions used were 30 cycles of 1 min of denaturation at 94°C and 2.5 min of elongation at 72°C for all reactions, with annealing for 1 min at 60°C (i*caA*), 59°C (*icaB*), 45°C (*icaC*), 59°C (*icaD*), or 59°C (IS*256*). After amplification in a T-personal thermocycler (Whatman Biometra GmbH, Göttingen, Germany), 5 μl of the PCR mixture was used in analysis for horizontal electrophoresis in agarose gel of up to 2% tris-borate-EDTA. A 100 bp ladder DNA molecular weight marker was used. Samples with positive amplification for the *A *and *D *fragments were analyzed for the presence of the insertion element IS*256*, amplifying the sequence by PCR (Table 1). *S. aureus *ATCC 29247 and *S. epidermidis *ATCC 35984 (RP62A) were used as negative and positive controls.

**Table 1:** Primer sequences for PCR used in this study.

Primer	Sequence (5'-3')	Product size (bp)	PCR conditions
*icaA *forward *icaA *reverse	GAC CTC GAA GTC AAT AGA GGT CCC AGT ATA ACG TTG GAT ACC	814	30 s 95°C 60 s 60°C 90 s 72°C
*icaB *forward *icaB *reverse	ATG GCT TAA AGC ACA CGA CGC TAT CGG CAT CTG GTG TGA CAG	526	60 s 95°C 60 s 59°C 90 s 72°C
*icaC *forward *icaC *reverse	ATA AAC TTG AAT TAG TGT ATT ATA TAT AAA ACT CTC TTA ACA	989	30 s 95°C 60 s 42°C 90 s 72°C
IS*256 *forward IS*256 *reverse	TGA AAA GCG AAG AGA TTC AAA GC ATG TAG GTC CAT AAG AAC GGC	1102	60 s 94°C 60 s 59°C 90 s 68°C
*icaD *forward *icaD *reverse	AGG CAA TAT CCA ACG GTA A GTC ACG ACC TTT CTT ATA TT	371	60 s 94°C 60 s 59°C 150 s 72°C

Only one strain of each infection episode was included for analysis.

### Statistical analysis

To compare differences between groups Mantel-Haenszel *x*^2 ^or Fisher exact test were employed, a value of p < 0.05 was considered to be significant. Statistical analysis was performed with SPSS for Windows software version 11.0.

## Results

During the study period, one hundred isolates from patients with diagnosis of device-related infections were included. The episodes were divided in 40 CVC-related bacteremias, 32 ependymitis, and 28 peritonitis. *S. epidermidis *was isolated in 45 episodes, *S. aureus *in 39 and diverse coagulase negative *Staphylococcus *(CoNS)non-*epidermidis *in 16 (*S. hominis, S. haemolyticus, S. lugdunensis, S. auricularis, S. warnerii *and *S. sciurii*). 33% of the isolates were oxacillin-resistant, 31% were resistant to amikacin, 26% to norfloxacin and 0% to vancomycin. According to the PFGE results, all the strains included corresponded to a different genotype.

**Figure 1:**
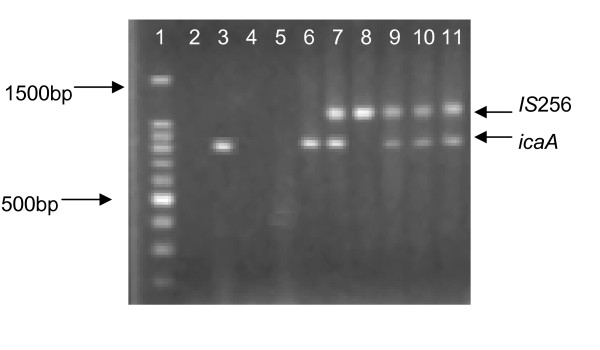
**Electrophoresis of PCR products with primers for IS*256 *and *icaA***. Lane 1, 100 bp molecular weight marker; lane 2, negative control; lane 3, 814-bp band from *icaA *positive control; lane 4 and lane 5, negative samples; lane 6, 814-bp band from *S. aureus *strain; lane 7, 1102-bp band (IS*256*) and 814-bp band (*icaA*) from *S. epidermidis *strain; lane 8, 1102-bp band from IS*256 *positive control; lane 9, lane 10 and lane 11, 1102-bp bands (IS*256*) and 814-bp bands (*icaA*) from *S. epidermidis *strains.

**Figure 2:**
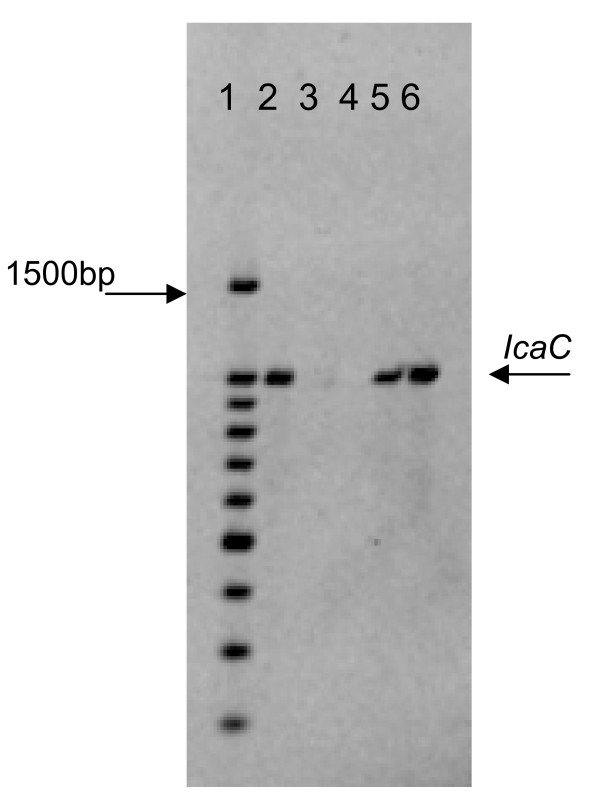
**Electrophoresis of PCR products with primers for i*caC***. Lane 1, 100 bp molecular weight marker; lane 2, 989-bp band from *icaC *positive control; lane 3, negative control; lane 4, negative sample; lane 5 and lane 6, 989-bp bands (*icaC*) from *S. epidermidis *strains.

**Figure 3:**
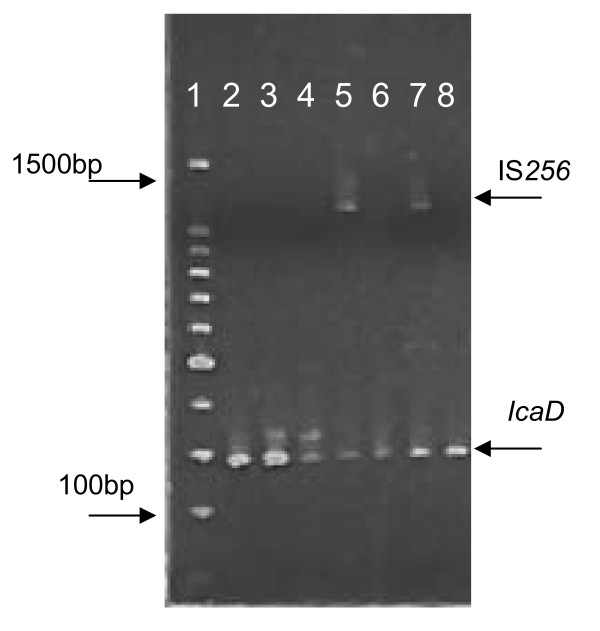
**Electrophoresis of PCR products with primers for IS*256 *and i*caD***. Lane 1, 100 bp molecular weight marker; lane 2, 371-bp band from i*caD *positive control; lane 3 and lane 4, 371-bp bands (i*caD*) from *S. haemolyticus *strains; lane 5, 1102-bp band (IS*256*) and 371-band (i*caD*) from *S. haemolyticus *strain; lane 6, 371-bp band (i*caD*) from *S. haemolyticus *strain; lane 7, 1102-bp band (IS*256*) and 371-band (i*caD*) from *S. haemolyticus *strain; lane 8, 371-band (i*caD*) from *S. haemolyticus *strain.

Biofilm production was confirmed in 22 strains by the Christensen assay, of these, 19 had a positive phenotype on the CRA plates. *IcaADBC *genes and the bacterial insertion sequence IS*256 *were detected in most of the strains, except for two strains of *S. auricularis*, one *S. warnerii *and one *S. sciurii*. 73.77% of CoNS and 79.5% of *S. aureus *isolates harbored the *icaD *gene; *icaA, icaB *and *icaC *genes were present in 27.8%, 21.3% and 9.8% of CoNS isolates (Table 2, figure 1). Only 4/39 *S. aureus *isolates were positive for *icaA+IcaD *genes but none for the bacterial insertion sequence. *IS*256-A+ *IS*256-D genes were detected in 21/45 *S. epidermidis *and 6/16 non-*epidermidis *CoNS. (figure 2, figure 3).

**Table 2:** *IcaADBC *genes and bacterial insertion element IS*256 *in *Staphylococcus *spp. isolates from device-related infections.

Microorganism	No. isolates	*IcaA*	*IcaB*	*IcaC*	*IcaD*	*IcaA *+ *IcaD*	IS*256-A*/IS*256-D*
*S. epidermidis*	45	23	11	4	39	22	21
*S. aureus*	39	4	0	0	31	4	0
*S. hominis*	6	4	0	0	6	4	3
*S. haemolyticus*	4	1	1	1	2	2	2
*S. lugdunensis*	2	1	1	1	1	1	1
*S. auricularis*	2	0	0	0	0	0	0
*S. warnerii*	1	0	0	0	0	0	0
*S. sciurii*	1	0	0	0	0	0	0

Therapeutic failure in episodes caused by an *IS*256-A+ *IS*256-D positive strain was 95.4% for *S. epidermidis*, RR 5.49 (CI 95% 2.24-13.44, p ≤ 0.0001), and 85.76% in CoNS isolates, RR 2.57 (CI 95% 0.97-6.80, p = 0.05). Although none *S. aureus *was positive for *IS*256-A + *IS*256-D, therapeutic failure was observed in 35.8%. (Table 3).

**Table 3:** *IcaAD *genes, bacterial sequence element and risk for therapeutic failure.

Microorganism (No. isolates)	*IcaAD *genes	Bacterial sequence element	Failure	Risk
*S. epidermidis* (45)	*icaA + icaD* 22	IS*256-A/* IS*256-D* 21	21/22 vs 4/23	RR 5.49 (2.24-13.44) p < 0.0001*
Non-*S. epidermidis *CoNS^&^ (16)	*icaA + icaD* 7	IS *256-A*/ IS *256-D* 6	6/7 vs 3/9	RR 2.57 (0.97-6.80) p = 0.05**
*S. aureus* (39)	*icaA + icaD* 4	IS*256-A/* IS*256-D* -	2/4 vs 13/35	RR 1.35 (0.46-3.93) p = 0.5**

^&^Coagulase-negative *Staphylococcus*. *Mantel-Haenszel square-chi. **Fisher-exact test.

## Discussion

Biofilm production has been clearly linked to infections in the presence of foreign bodies for various decades [26], it has been demonstrated *in vivo *tests with laboratory animals and *in vitro*, that biofilm hampered thorough penetration of the antimicrobial and the concentrations require to eradicate biofilm-producing strains are higher than those required to eradicate strains that did not produce biofilm [27,28].

In this study, as reported by other authors, it was found that the genes of the *Ica *operon frequently appeared in strains of *Staphylococcus epidermidis *[4,6,29]. The number of *S. aureus *isolates with a positive biofilm phenotype or producing biofilm was low (4/39), and in none of them the bacterial insertion element IS*256 *was present. Experimental studies have also shown that biofilm formation is possible in *icaADBC *operon *S. aureus *mutants [30], but we did not find *S. aureus *negative for the operon that was a biofilm producer. *S. epidermidis *strains with the genetic determinant for biofilm formation were clearly associated to a high risk of therapeutic failure, which was not corroborated with non-*epidermidis *CoNS and *S. aureus*, perhaps due to a smaller number of collected isolates.

As previously stated, adhesion to the plastic device could be a factor linked to weaker expression of symptoms of infection, due to the existence of a smaller number of circulating bacteria, most of them remaining inside the catheter. This mild presentation could support the non-standard recommendation to preserve the medical device in patients with bacteremia. This is necessary in some cases due to the critical condition of the patient. However, the risk of failure to proper antimicrobial treatment is so high, that this conduct should be avoided and the CVC removed as soon as possible, even if a negative *icaA +IcaD *isolate is detected.

The present work has several limitations. Infections included are difficult to compare to elicit general recommendations, in particular for the ependymitis and peritonitis episodes. The *ica *operon genes have been widely described in *Staphylococcus epidermidis *and *Staphylococcus aureus*, several authors have found similarity in other CoNs species [6,7] but results cannot be extended to all pathogenic species. Recently biofilm formation has been described in detail in *S. haemolyticus *isolates [31]. Phenotypic variation as well as clonal lineage must be included in future studies to develop alternative therapeutic strategies.

## Conclusions

The presence of *icaA/D *genes along with the sequence element *IS*256 was associated with therapeutic failure in most CoNS infections, even though its absence in *S. aureus *isolates does not ensure therapeutic success.

## Competing interests

The authors declare that they have no competing interests.

## Authors' contributions

BDH participated in data collection, analysis, interpretation, and literature research; FSS performed a critical review; BLM participated in data collection and laboratory assays; LPB helped to review the manuscript; and GMN conceived the study and participated in design, analysis, supervision of laboratory assays and corrected the manuscript. All authors read and approved the final manuscript.

## Pre-publication history

The pre-publication history for this paper can be accessed here:

http://www.biomedcentral.com/1471-2334/10/68/prepub
